# Research models to study lewy body dementia

**DOI:** 10.1186/s13024-025-00837-w

**Published:** 2025-04-23

**Authors:** Suelen Lucio Boschen, Aarushi A. Mukerjee, Ayman H. Faroqi, Ben E. Rabichow, John Fryer

**Affiliations:** 1 Department of Neuroscience, Mayo Clinic Jacksonville, 4500 San Pablo Rd, Jacksonville, FL 32224 USA; 2 Department of Neurosurgery, Mayo Clinic Jacksonville, 4500 San Pablo Rd, Jacksonville, FL 32224 USA; 3 Mayo Clinic Graduate School of Biomedical Sciences, Mayo Clinic, 200 First St. SW, Rochester, MN 55905 USA; 4 Translational Genomics Research Institute, 445 N 5th St, Phoenix, AZ 850054 USA

**Keywords:** Synucleinopathy, Animal model, Cell culture, Dementia with lewy body, Parkinson’s disease dementia, Alpha-synuclein, Beta-amyloid, Tau

## Abstract

Lewy body dementia (LBD) encompasses neurodegenerative dementias characterized by cognitive fluctuations, visual hallucinations, and parkinsonism. Clinical differentiation of LBD from Alzheimer’s disease (AD) remains complex due to symptom overlap, yet approximately 25% of dementia cases are diagnosed as LBD postmortem, primarily identified by the presence of α-synuclein aggregates, tau tangles, and amyloid plaques. These pathological features position LBD as a comorbid condition of both Parkinson’s disease (PD) and AD, with over 50% of LBD cases exhibiting co-pathologies. LBD’s mixed pathology complicates the development of comprehensive models that reflect the full spectrum of LBD’s etiological, clinical, and pathological features. While existing animal and cellular models have facilitated significant discoveries in PD and AD research, they lack specificity in capturing LBD’s unique pathogenic mechanisms, limiting the exploration of therapeutic avenues for LBD specifically. This review assesses widely used PD and AD models in terms of their relevance to LBD, particularly focusing on their ability to replicate human disease pathology and assess treatment efficacy. Furthermore, we discuss potential modifications to these models to advance the understanding of LBD mechanisms and propose innovative research directions aimed at developing models with enhanced etiological, face, predictive, and construct validity.

## Background

Lewy body dementia (LBD) is an umbrella term for neurodegenerative dementias, including Parkinson’s disease dementia (PDD) and dementia with Lewy bodies (DLB). These conditions are clinically characterized by cognitive fluctuations, visual hallucinations, sleep behavior disorders, and parkinsonism. Clinical diagnosis of LBD is challenging due to the similarities of cognitive and behavioral symptoms present in Alzheimer’s disease (AD), which remains the most prevalent type of dementia in the elderly population [1]. About 25% of dementia cases are neuropathologically diagnosed as LBD due to the presence of α-synuclein (αsyn) aggregates in cytoplasmic structures known as Lewy bodies (LB) and Lewy neurites (LN), accumulation of hyperphosphorylated tau in neurofibrillary tangles (NFT), and of β-amyloid (Aβ) into amyloid plaques [2]. The presence of Lewy pathology (LP) is a key pathological feature of PD, while NFTs and Aβ plaques are hallmarks of AD. Thus, LBD represents comorbid pathologies of PD and AD, with about 50% of LBD patients harboring co-pathologies of αsyn, Aβ, and tau accumulation [3, 4]. 

The primary clinical distinction between PDD and DLB lies in the timing of dementia symptoms relative to the onset of parkinsonism. Specifically, approximately 83% of PD patients develop dementia symptoms after one year of parkinsonism onset classifying them as PDD patients [5]. Conversely, patients who exhibit parkinsonism within or after one year of cognitive or behavioral symptoms are diagnosed with DLB. Notably, around 25% of DLB patients never develop parkinsonism [6]. 

Much of our current understanding of these neurodegenerative diseases has been achieved through research models that replicate certain hallmarks of the diseases enabling controlled investigation of specific pathophysiological mechanisms and potential treatments [7]. As with most models of disease, it is rare to find a disease model that recapitulates the entirety of the etiological, clinical, and pathological features of the disease. While numerous animal and cellular models that mimic human degenerative diseases to a certain extent exist for PD and AD, the pathogenic processes that differentiate LBD from PD and AD remain poorly understood due to the lack of mixed-pathology models with sufficient etiological, face, predictive, and construct validity.

In this review, we describe some of the most commonly used research models for PD and AD research focusing on their ability to represent the true nature of these diseases, replicate human pathological features, and evaluate the effectiveness of new therapies. Additionally, given that LBD may fall within a spectrum of ataxias and dementias, we propose new research directions to study the mechanisms of LBD based on adaptations of existing PD and AD models.

### Parkinson’s disease

Parkinson’s disease is the most common movement disorder and the second most common neurodegenerative disorder after AD. Prodromal non-motor symptoms, such as changes in sleep and olfaction, depression, and gastrointestinal issues like constipation, appear long before the clinical diagnosis of PD highlighting the progressive nature of the disorder. Clinical motor features that prompt diagnosis include tremors, postural instability, rigidity, and bradykinesia. While bradykinesia is always present, approximately 20% of PD patients do not have tremors which depicts the variability of symptom presentation among patients [8–10]. For this reason, clinical diagnosis of PD depends on the presentation of bradykinesia along with at least one of the characteristic motor symptoms [11]. 

Neuropathologically, PD is characterized by the loss of dopaminergic neurons in the substantia nigra *pars compacta* (SNc) which results in decreased levels of dopamine (DA) in the striatum and gives rise to the classical motor deficits. Additionally, abnormal deposition of hyperphosphorylated, aggregated αsyn, various other proteins, lipids, and organelles culminates in intracytoplasmic neuronal inclusions called LB and LN [12]. Lewy pathology initially occurs in cholinergic and monoaminergic brainstem neurons and in the olfactory bulb and is thought to occur 10–20 years prior to motor symptoms [13]. As the disease progresses to more advanced stages, LP is found in limbic and cortical areas which can indicate the occurrence of cognitive dysfunction more commonly associated with PDD [13, 14]. 

The precise etiology of PD remains unclear, although several risk factors are known to contribute to sporadic idiopathic PD development, including advanced age, traumatic brain injury, and exposure to environmental factors such as paraquat and rotenone [12, 15]. Genetic forms of PD represent 5–10% of all cases including mutations in the *GBA* gene that encodes glucocerebrosidase and accounts for 5–15% of PD patients, as well as mutations in the *LRRK2* gene encoding for leucine-rich repeat kinase 2, the *SNCA* gene for αsyn, and the *PRKN* and *PINK1* genes for parkin and pink. Large genome-wide association studies (GWAS) confirmed that some of these genes are also implicated in sporadic PD due to their involvement in a set of molecular pathways that can trigger a neuropathology similar to PD. These pathways include αsyn proteostasis, mitochondrial function, oxidative stress, calcium homeostasis, axonal transport, and neuroinflammation [15, 16]. 

Although there has been remarkable progress towards characterizing and developing experimental models of PD in recent years due to our increased understanding of the etiopathogenesis and manifestation of the pathology in human disease, there is still no one model that encompasses the multiple coexisting cellular and behavioral changes observed in the disease. Rodents, non-human primates (NHP), and cell-based models each have distinct advantages and limitations that offer new opportunities for researchers. However, investigators often have to select the most suitable model for the specific scientific question being asked.

### Animal models of PD

Non-mammalian models of PD, including *Caenorhabditis elegans* and *Drosophila melanogaster*, share approximately 80–85% conversed molecular pathways and cellular processes with humans that can be explored to study PD pathogenesis [17]. These model organisms can breed in large numbers, have a short generation time, and require relatively low maintenance costs. The main limitation is that *C. elegans* and *D. melanogaster* do not express αsyn, although transgenic overexpression of αsyn can be achieved to further investigate its relationship with PD-associated gene mutations and signaling and proteasomal pathways [17]. Additionally, results from non-mammalian small models need to be validated in mammalian animal models and human neuronal cell cultures, as will be discussed in the next sections.

Rodents are the species most commonly used as PD models for several reasons including the ease of care, relatively low costs for maintenance, less ethically problematic than NHPs, and availability of transgenic mouse strains [18]. Additionally, rodents show a significant degree of human homology regarding the organization of cortico-basal ganglia-thalamocortical loops and their corresponding motor and affective functions [19, 20] coordinating complex behaviors that can be studied through a series of behavioral tests. Non-motor symptoms related to sleep, motivation, and risk avoidance can also be modeled as an early stage of PD, as can studies addressing the functionality of peripheral organs (in particular, bladder, heart, and gastrointestinal tract) in the setting of experimental parkinsonism or synucleinopathy [21, 22]. 

NHP models account for about 10% of PD research [18]. NHPs, particularly macaque monkeys, are closely related to humans genetically and physiologically and offer specific advantages regarding the phenomenology and mechanisms of disease [23, 24]. This allows for the quantification of Parkinsonian and dyskinetic features in NHPs using principles of the Unified Parkinson’s Disease Rating Scale (UPRS) similar to those applied in humans, streamlining the translational path from the lab to the clinic [25]. The main disadvantages of NHP models are the high costs associated with animal care, specialized and extensive labor, the necessity of highly specialized housing facilities, and strict ethical considerations [26]. 

Beyond animal species, PD models differ based on the challenge, injection area, administered dose and the dosing paradigm (e.g., acute vs. chronic treatments). For example, the synthetic dopamine derivative 6-hydroxydopamine (6-OHDA) was one of the first chemical challenges used to model PD in rodents [27]. The addition of one hydroxyl group to the structure of DA confers toxicity to catecholaminergic neurons by rapidly oxidizing and producing reactive oxygen species (ROS) by monoamine oxidase (MAO) catabolism. ROS build-up inhibits complex 1 of the mitochondrial respiratory chain and culminates in neuronal dysfunction and death [26]. 6-OHDA can be directly injected in the SNc, medial forebrain bundle (MFB), or striatum to selectively damage catecholaminergic neurons in the nigrostriatal system recapitulating loss of DA transmission found in PD and producing deficits in motor function that vary in severity according to the extent of DA lesion [28]. Bilateral lesions from 6-OHDA injections in the MFB produce dose-dependent, extensive retrograde degeneration of DA neurons in the SNc that can be quite severe leading to bradykinesia, changes in gait and nociception, cognitive deficits, depressive-like behavior, and enteric nervous system dysfunction [29]. Unilateral injections are more commonly used to study forepaw asymmetry and rotational motor behavior (Table 1) [30]. Essentially, the rodent 6-OHDA is recommended for screening of symptomatic therapies, levodopa-induced dyskinesia, and for studies of motor and non-motor symptoms, with the latter achieved following partial striatal injections of low doses of 6-OHDA.

**Table 1:** Animal models of Parkinson’s disease

Model	α-synuclein pathology	Dopaminergic (TH+) neurodegeneration	Motor deficits	Cognitive/Behavioral deficits	References	
Toxin Models of Parkinson’s disease						
6-OHDA (mice, rats)	Not present	Nigra: Within 12 h of injection, with progressive fiber degeneration during the following 7–10 av days Medial frontal lobe: 3 to 5 weeks post-injection Striatum: 24 h after injections which progress up to 3 weeks	Appears 1 week post injection and severity vary with extension of DA neurodegeneration	Behaviors deficits can appear 2–3 weeks after injection.	[231–233] [234]	
MPTP (mice, rats, NHPs)	NHP may develop αsyn inclusions reflective of an early stage of PD at 1-month post-injection	Acute: 12 h in mice Chronically: 3 weeks for degeneration of the nigrostriatal pathway in mice 42 + days in rats 1-month post-injection in monkeys. Mice and NHP are treated systemically. Rats require intra-cranial injection.	Rotarod deficits appear 2 weeks after injection. Rats and mice display mild symptoms. NHPs display parkinsonism-like symptoms.	Increase in anxiety-like behavior and impairment of object recognition in rats. NHP demonstrates impairment across multiple cognitive test 3–5 weeks after administration.	[37, 235–237] [238] [239, 240] [241]	
Rotenone (mice, rats)	αsyn accumulation in the SNc present 9 months after rotenone exposure in rats In mice αsyn accumulation occurs two weeks after exposure in the striatum and SNc	Significant loss of dopamine neurons 3 months after rotenone exposure In mice two weeks after exposure experienced substantial loss of dopamine neurons in the SNc after 2 weeks	Acute motor deficits present within 3–5 days after injection. More prevalent motor deficits after 3 months in rats	Behavioral symptoms may occur in rats after 4 months post-exposure in rats. Cognitive deficits can occur in mice 3 weeks after exposure to rotenone Gastrointestinal dysfunction may also be present in rats 4 weeks after exposure.	[44–46, 242–244] [245] [246]	
**Non-toxin models of Parkinson’s disease**						
AAV-αsyn (mice, rats, NHPs)	Present– αsyn overproduction and aggregates. LB-like inclusions in NHPs after 4 months.	Rodents: Degeneration of neurons in the nigrostriatal pathway at 3–4 weeks after injection NHP: Degeneration of dopamine neurons takes 4 months in the nigrostriatal pathway Extension of lesion varies according to promotor type, capsid serotype, wild-type αsyn or mutated αsyn, etc.	Motor deficits 7 weeks post-injection in rodent models (staircase, stepping test, and rotarod) Motor deficits appear at 6 months in NHP	Depressive-like phenotype in the swim test 3 weeks post injection.	[62, 66, 247, 248] [249]	
PFF (mice, rats)	Present– αsyn aggregates and LB-like inclusions in the nigrostriatal pathway, thalamus, and occipital cortex Rats show αsyn accumulation in the nigrostriatal pathway in 2 months, and the amygdala in 6 months post-injection,	Mice: 60 days post-injection in the nigra and 90 days post-injection Rats: Degeneration of dopamine neurons in the nigra occurs 4 to 6 months after injection in rats	Motor deficits are present approximately 60 days post-injection in mice.	Not present.	[72, 73] [250–253]	
M83 Transgenic mice (A53T αsyn)	Present after 20 months	Dopamine degeneration in the nigra between 8–16 months of age	Movement is impaired by 8 months of age.	Sensorimotor deficits at 1–2 months Cognitive deficits present at 6 months	[49, 50, 254]	
Line 61 mice	Present	Present	Present	Present	[53, 54]	
BAC-*LRRK2*-R1441G transgenic mice	Not present	Dopamine loss in the SNc occurred at age 9–10 months old	Motor deficits observed at 10–12 months	Not present	[255] [256]	
BAC*-SNCA*-A30P transgenic mice	αsyn overproduction at 3 months in the SNc and at 6 months in the STR.	Not present	Mild rearing impairment at 12 months	Not present	[57] [257]	
BAC-*SNCA*-OVX transgenic mice	Overproduction in the midbrain at 3 months of age.	Loss of dopamine neurons in the SNc at 18 months of age.	Motor symptoms present at 18 months of age.	Gastrointestinal symptoms are present.	[58, 258]	
BAC-*SNCA*^A53T/−^ transgenic mice	Overproduction after one month post injection in the SNc, Striatum, olfactory bulb and cerebral cortex	Acute loss at 1 month post injection Loss of dopamine neurons at 18 months of age	Present at 6 months	Rapid eye movement at 5 months of age. Hyposmia at 9 months of age	[51, 59]	

Unlike 6-OHDA, 1-methyl-4-phenyl-1,2,3,6-tetrahydropyridine (MPTP), an organic compound with analgesic properties, can be delivered systemically due to its lipophilicity. After crossing the blood-brain barrier, MPTP targets astrocytes and is metabolized by MAO-B into 1-methyl-4-phenylpyridinium (MPP+) which is a structural analog to DA. Once DA neurons uptake MPP+, the toxin inhibits mitochondrial complex I of the electron transport chain, leading to excessive production of ROS and oxidative stress, which triggers degeneration of DA neurons [26]. Acute, subchronic, or chronic regimens of MPTP intoxication have been largely used to induce selective dopaminergic degeneration and motor deficits in NHPs [24], minipigs [31], and mice [32]. Rats are resistant to systemic administrations of MPTP due to their capacity for vesicular sequestration of this toxin [33], although direct SNc injection of MPTP caused DA degeneration and motor and cognitive deficits comparable to an early phase of PD [34]. Chronic and low systemic doses of MPTP in NHPs has been proposed as a progressive PD model because it closely models human PD motor symptoms, including bradykinesia and rigidity [35], levodopa-induced dyskinesia [36], and cognitive impairment [37]. The NHP MPTP PD model is one of the most recommended models to test potential neuroprotective (e.g., stem cell therapies) and symptomatic therapies [38, 39]. 

Rotenone is a natural compound found in plants and has been used as a pesticide. The rotenone-induced PD model is typically established in rats but can also be done in mice, fish, and invertebrates [40–42]. Chronic, low doses of this toxin readily penetrate the blood-brain barrier (BBB), inhibit mitochondrial complex I, reduce glutathione levels, and trigger oxidative stress, culminating in the degeneration of monoaminergic neurons, especially DA neurons [43]. The rotenone rat model exhibits motor symptoms similar to those of human PD, such as slow gait, stiff movements, limb tremors, reduced motor activity, and lethargy [44], and depressive-like behavior during forced swimming and sucrose preference tasks [45]. This model is also capable of exhibiting LB-like inclusions in the gastrointestinal tract [46], and accumulation of αsyn in the cytoplasm of surviving neurons [47, 48]. 

The toxin-induced PD models are effective tools to investigate the symptomatic therapeutic potential of new drugs but lack the ability to replicate the progressive pathogenesis of PD. Transgenic models expressing human αsyn enhance the pathological manifestations of the disease and allow for studies focusing on the investigation of PD pathophysiological mechanisms. The M83 transgenic mouse expresses human αsyn with the A53T mutation under the mouse prion protein promoter resulting in transgene expression in the cerebral cortex, spinal cord, and cerebellum [49]. Motor impairments in M83 homozygous mice are accelerated (16 months) compared to hemizygous mice (22–28 months) highlighting the influence of expression levels on pathogenesis. Additionally, M83 mice develop age-dependent intracytoplasmic inclusions of αsyn in neurons that were shown to be seed-competent making it an ideal model for synuclein transmission and propagation studies [50]. Other transgenic mouse models featuring overexpression of A53T mutation reflect the pathological manifestations of αsyn, and exhibit pronounced neurodegeneration and motor deterioration, along with non-motor symptoms, such as sleep and olfactory dysfunction [51]. However, the A30P transgenic mouse model is capable of manifesting non-motor symptom disorders characteristic of early PD in humans such as impairment in visual acuity, olfactory dysfunction, and mood abnormalities [52]. 

The Line 61 mouse model overexpresses human αsyn under the murine Thy-1 promoter which has the highest levels of expression in the neocortex, hippocampus, olfactory bulb, thalamus, colliculus, substantia nigra, and brainstem [53]. At 14 months, Line 61 mice exhibit progressive features of sporadic PD such as approximately 40% reduction in striatal DA, 17% reduction of striatal TH, an early phase of locomotor hyperactivity (4–5 months), and a late phase with consistent motor deficits. Non-motor function is also affected by changes in circadian rhythm and gut function preceding motor impairments, suggesting an early impact of the αsyn transgene in areas outside the nigrostriatal system, akin to the prodromal phase of human PD [54, 55]. One of the caveats of this model however is that the transgene is located on the X chromosome, which precludes it as a useful model to investigate sex differences due to X inactivation in males [54]. 

Bacterial artificial chromosome (BAC) animal models are an alternative to using heterologous gene promoters allowing for an endogenous transgene expression profile under the control of native promoters. Most of the *LRRK2* transgenic mice models failed to recapitulate important PD hallmarks. For example, the BAC-LRRK2-R1441G transgenic mice show motor deficits and axonal pathology in the striatum, but no DA neuronal loss and αsyn aggregation [56]. Similarly, *SNCA* A30P BAC mice expressing the A30P αsyn mutation (or their wild-type littermates) do not show loss of midbrain catecholaminergic neurons, LB-like aggregates, but demonstrate reduced DA release in the dorsal striatum [57]. Other *SNCA* BAC mice models present more PD-like features which can be further explored to elucidate the biochemical and functional changes induced by human αsyn. The SNCA-OVX mouse model was generated using the entire human *SNCA* locus with native promoter and regulatory elements to express αsyn at disease-relevant levels with a correct spatiotemporal expression profile [58]. These mice present early changes in DA transmission and age-dependent loss of nigrostriatal DA neurons and motor impairment [58]. The BAC-*SNCA*^A53T/−^ mice can be used as a model of prodromal PD because they develop characteristic non-motor symptoms, such as rapid eye movement (REM) sleep behavior disorder at 5 months old and hyposmia at 9 months old despite presenting a mild age-dependent DA neurodegeneration phenotype. Importantly, the emergence of behavior changes correlates with the accumulation of αsyn and phosphorylated αsyn in region-specific brain regions such as lower brainstem and olfactory bulb [51]. Recently, Okuda and colleagues demonstrated that intra-striatal injection of mouse αsyn pre-formed fibrils (PFF) in BAC-*SNCA*^A53T/−^ induces a more severe αsyn pathology than in mice expressing wild-type human αsyn, as well as more severe than the intra-striatal injection of human αsyn PFF. BAC-*SNCA*^A53T/−^ mice injected with mouse PFF present approximately 40% loss of tyrosine hydroxylase-positive neurons in the SNc and significant motor dysfunction at 2 months post-injection [59]. Kikuchi et al. used an αsyn BAC transgenic mouse model of αsyn overexpression [60] to show that transplantation of PD patient-derived induced pluripotent stem cells (iPSC) DA progenitors in 6 months-old mice does not cause an accumulation of pathological αsyn [61]. Thus, the speed of progression and severity of αsyn pathology depends on several factors, including the variants of αsyn endogenously expressed, the levels of expression, and the types of αsyn PFF used.

Recombinant adeno-associated viral vector (AAV) can be used as a vehicle to deliver a specific PD-associated gene and induce its overexpression. Local SNc injections of wild-type or mutated (e.g., A53T or A30P) αsyn can lead to efficient transduction of TH-positive neurons in rats with progressive development of LN- and LB-like inclusions, 30–80% DA cell loss, 40–50% reduction in DA transmission, and reduced motor function [62]. Higher levels of DA degeneration, motor impairment, or αsyn aggregates can be achieved depending on several factors such as the promotor type, insertion of a transduction enhancer [63, 64],, and variations in capsid serotypes [65]. Similarly, AAV-mediated overexpression of wild-type and A53T αsyn in marmosets caused LN and αsyn aggregates in the soma and 30–60% DA neuronal loss in the nigrostriatal pathway [66]. Overexpression of human αsyn using AAVs was also achieved with intra-SNc injections in mice. These mice developed a mild and slow-progressive phenotype with about 25% DA degeneration at 6 months post-injection [67]. Notably, for AAV-mediated overexpression of αsyn, special attention must be given to the AAV serotype as its tissue tropism can dramatically affect transduction efficiency and off-target effects. For example, AAV1, AAV2, AAV6, AAV8 and AAV9 have good brain tropism. Newer variants, such as rh8 and rh10, might be even more specific to the brain with lower tropism to other body tissues [68]. 

More recently, PFFs of monomeric recombinant αsyn can be generated and directly injected into the SNc to seed endogenous αsyn to misfold and form LB-like cytoplasmic inclusions. Specific guidelines are strongly recommended for PFF modeling of PD in animals [69]. The molecular size of PFFs is a crucial determinant of efficacy, with optimal modeling ranging from 29 to 49 nm [70]. In addition, it is recommended to employ an injection concentration of 1 µg/mL of PFF [71]. Both mice and rats develop LB-like inclusions mostly in the area of injection with spread αsyn aggregates to other brain areas such as the cortex, olfactory bulb, amygdala, thalamus, and striatum. Slow, progressive degeneration of DA neurons can also be observed [71–76]. PFF inoculation can also start an immune response as MHCII-positive cells are found in the brain in greater magnitude during aggregation stages that precede degeneration [77]. Recently, Uemura and colleagues demonstrated that dorsal striatum injection of αsyn aggregates amplified from patient-derived LB (ampLB) induces pathologies similar to those of LBD subjects. Moreover, the authors showed that modeling PD with αsyn PFF or ampLB produces important differences associated with their intrinsic biological activity, such as seeding activity, latency in inducing pathology, distribution of pathology, morphology of neuronal inclusions, and cell-type preference [78]. 

### Cellular models of PD

Several cellular models have been developed to study the pathogenesis of PD and to identify therapeutic targets. Immortalized cell lines are highly advantageous because of their relatively inexpensive and straightforward maintenance and continuous proliferation, which allows for a broad range of applications with flexible experimental designs and methodologies. Immortalized cells can also be transfected with wild-type or mutated αsyn to stably overexpress this protein making them useful for studying PD pathology [79]. 

One of the immortalized cell lines most commonly used in PD research is the human neuroblastoma cell line SH-SY5Y (Table 2). The SH-SY5Y cells are superior to other cell lines commonly used in neurodegenerative research, such as neuroglioma cells H4 and human embryonic kidney 293 (HEK293), because undifferentiated SH-SY5Y cells present a neuron-like structure expressing immature neuronal markers. Additionally, SH-SY5Y cells can be differentiated in a catecholaminergic neuron-like phenotype [80, 81] and reproduce certain PD phenotypes such as LB-like inclusions following PFF treatment [82]. The disadvantages of this cell line include possible alterations in the differentiation fate, viability, growth performance, metabolic properties, and genomic stability due to its neoplasmic origin. Additionally, the lack of standardized source, maintenance, and differentiation protocols produces inconsistent experimental outcomes, making data reproducibility a big challenge when working with SH-SY5Y cells [83]. The Lund human mesencephalic (LUHMES) cell line derives from a healthy 8-week-old human mesencephalic embryonic tissue and is immortalized by the insertion of a *myc* oncogene under the control of a tetracycline-responsive promotor [84]. These cells can be more consistently differentiated into a dopaminergic neuron-like phenotype showing mature neuronal markers, long neuronal processes, and electrical activity similar to those of dopaminergic neurons. Such characteristics allow for higher throughput cell-based assays than SH-SY5Y cells [85, 86]. LUHMES cells have also been used in the development of a spheroid 3D model composed of neurons, astrocytes, and oligodendrocytes undergoing myelination and synaptogenesis with rapid maturation (~ 25 days) and reasonable longevity (~ 60 days) [87, 88]. 

Non-neuronal immortalized cell lines, such as HEK293 and H4 lines, can be easily transfected with transient and constitutive overexpression of human wild-type or mutated. These biosensor reporter cell lines are useful in vitro seeding assays because they allow tracking of αsyn aggregates that form within a relatively short time (~ 24 h), according to the specific paradigm [89–92]. Fluorescent labeling or protein complementation assays (PCA) are used in HEK293 [93] and H4 cells [94] to detect and quantify αsyn/αsyn interactions [95] Despite the lack of dopaminergic phenotype, these cell lines are easy to culture and suitable for high-throughput screens of drugs effective against the toxic effects of αsyn [96]. The cell-free seeding assay, known as real-time quaking-induced conversion (RT-QuIC) assay, enables the detection of αsyn aggregation based on the amplification of αsyn aggregates induced by pathogenic seeds present in the analyzed sample, such as the cerebrospinal fluid (CSF) [97, 98], brain homogenate samples [99–101],, and skin [102, 103]. This assay has critical importance in the identification of pathological seeds and is currently under intense study to be used as a biomarker for the diagnosis of synucleinopathies [103, 104]. A more detailed review can be found in [105].

Primary culture of murine embryonic or early postnatal neurons is a good alternative to the inherent limitations of immortalized cell lines. These cultures typically result in a mixture of different types of neurons including around 10% of dopaminergic neurons [106]. The rapid differentiation into neurons forming neurites and synapses allows for a broad range of mechanistic studies on the pathogenesis of αsyn in cellular organelles and biochemical pathways [107]. Primary cultures also offer the versatility of adapting differentiation protocols to allow a mixed culture of neurons and glial cells, such as microglia, more closely capturing the influence of immune system modulation in PD pathology [108, 109]. Limitations of primary cultures include lower translational properties compared to the use of cell lines of human source, difficult genetic manipulation often requiring viral transduction, and reduced yield for subsequent assays [110]. 

Somatic cells from PD patients can be reprogrammed iPSC capable of being re-differentiated into any cell type, including neuronal cells while continuing to exhibit genetic PD phenotype [111, 112]. Patient-derived iPSCs are an excellent disease-in-a-dish model with high translational capabilities enabling in vitro clinical trials that enhance the outcome predictability of actual clinical trials and open up possibilities of individualized treatment [113, 114]. Several molecular mechanisms associated with neuronal dysfunction in PD have been demonstrated in PD patient-derived iPSCs. Accumulation and aggregation of αsyn have been observed in iPSC-derived neurons from patients carrying mutations, duplications, or triplications in the *SNCA* gene [96, 115]. Mitochondrial dysfunction has been reported in iPSCs of PD patients carrying mutations in *GBA*,* PINK1*,* PRKN*, and *LRRK2* genes [116–120]. Oxidative stress, proteasomal impairment, axonal degeneration, and even iPSC models of sporadic PD have also been reported [121–124]. 

Three-dimensional (3D) brain organoids are formed from a mixture of human iPSCs-derived neurons and glial cells cultured using artificial matrices that resemble the extracellular matrix (e.g., matrigel) and can mature in a more physiologically relevant microenvironment, closely mimicking the complexity of cellular interactions in the brain. Becerra-Calixto and colleagues built a human midbrain-like organoid model using iPSCs from a PD patient carrying *SNCA* gene triplication. They reported LB-like cytoplasmic inclusions, increased apoptotic markers, and loss of DA neurons [125]. Remarkably, the first organoid model of sporadic PD, made from peripheral blood mononuclear cells of PD patients, was recently reported to maintain differences in the expression of early and late neuronal markers relative to organoids derived from healthy volunteers [126], indicating the valuable utility of such models in the study of molecular pathways involved in familial and sporadic PD. However, large-scale studies on patient-derived organoids remain more laborious and expensive than other in vitro models requiring written informed consent and approval from ethics committees. Nonetheless, increasing investments in technological approaches have allowed for the development of a high-throughput robotic microfluidic bioreactor system (Pelican) that adopts automation of cell culture protocols for more reproducible cellular differentiation, proposing better standardization of protocols between laboratories [127]. 

Direct neuronal reprogramming, by which a neuron is formed via direct conversion from a somatic cell without going through a pluripotent intermediate stage, allows the possibility to generate patient-derived neurons that maintain the aging and epigenetic signatures of the donor [128, 129]. Direct reprogramming of dermal fibroblasts from idiopathic PD patients into induced dopaminergic neurons (iDANs) showed impairments in stress-induced autophagy processes that were not present in age and sex-matched control iDANs and their parental dermal fibroblasts [130]. The study from Drouin-Ouellet and colleagues provides a novel, cost-efficient, and less labor-intensive model than the iPSC-modelling of idiopathic PD which maintains disease subtype identity and donor’s age, reflecting pathological changes as early as 25 days of culture [130]. For a detailed review of the comparisons of iDANs and iPSCs for PD, see [131].

**Table 2:** Cellular models of Parkinson’s disease

Cell Type	Characteristics of αsyn expression	αsyn aggregates	Dopaminergic-like cell	References
SH-SY5Y (neuroblastoma cells)	Endogenous human αsyn	May be induced by PFF, rotenone, or AAV-αsyn treatment.	Can be differentiated into catecholaminergic neurons	[80, 81, 83]
LUHMES (mesencephalic embryonic human cells)	Endogenous human αsyn	May be induced by PFF, rotenone, or AAV-αsyn treatment.	Can be differentiated into dopaminergic neurons	[84–86, 132]
Murine primary neurons	Endogenous murine αsyn	May be induced by PFF, rotenone, or AAV-αsyn treatment.	Mixture of different types of neurons (~ 10% are dopaminergic neurons).	[107, 108, 133]
iPSC (patient-derived induced pluripotent stem cells)	Endogenous or overexpression of αsyn depends on gene mutation or multiplication.	May be spontaneously present according to *SNCA* gene mutation or multiplication.	Can be differentiated into dopaminergic neurons	[114] [113, 116] [115]
iDANs (patient-derived induced dopaminergic neurons)	Endogenous expression of αsyn according to patient’s phenotype	Not observed	Yes	[130]
Brain organoids (LUHMES cells or patient-derived iPSC)	Endogenous or overexpression of αsyn depends on gene mutation or multiplication.	May be spontaneously present according to *SNCA* gene mutation or multiplication.	Mixture of different types of neurons and glial cells.	[88, 125] [126] [127]

### In Silico models of PD

The landscape of in silico models of PD is rapidly evolving, reflecting advances in computational biology, systems neuroscience, and artificial intelligence. These models aim to simulate various aspects of PD, including molecular pathogenesis, neural circuitry dysfunctions, and clinical outcomes.

Mettai and colleagues used a molecular docking study combined with an ADME analysis to clarify the bonding modes and affinity rates between the active site residues of MAO-B and a new class of MAO-B inhibitors to predict the drug-likeness properties of the best ligands. They successfully generated two new MAO-B inhibitors with predicted good bioavailability and high levels of gastrointestinal absorption [134]. Preclinical testing in in vitro and in vivo models are necessary to confirm these results. The Caulfield lab also provided insights into PD pathogenesis through the application of molecular dynamics simulations (MDS). They characterized hyperactive variants of parkin, an E3 ubiquitin ligase that mediates mitophagy, and elucidated different activation mechanisms for each hyperactive variant. This opens avenues for novel studies targeting parkin’s structure for potential therapeutic designs [135]. The use of quantitative systems pharmacology (QSP) frameworks integrates multi-scale data to predict drug effects and optimize precision medicine. These tools are essential for developing both symptom-modifying and disease-modifying therapies, which remain a challenge due to the high failure rates in clinical trials [134, 136]. 

Advanced artificial intelligence (AI) models, including deep neural networks, are used to simulate brain network changes, predict disease progression, and analyze large datasets for diagnostics. For example, deep convolutional neural networks can model structural and functional neural degeneration, helping researchers understand disease dynamics and potential interventions [137]. This is particularly important in the context of recent systems biology models integrating human genetic, transcriptomic, and proteomic data to link genetic variants with PD pathology, enhancing the discovery of novel therapeutic targets [136]. AI-driven PD research focused on human data has the potential to partially replace or optimize in vitro and in vivo models of PD, providing direct clinical relevance and applications. For example, the efficacy and toxicity of new therapeutics can be predicted through AI models of pharmacokinetics and pharmacodynamics, reducing the need for extensive animal testing. Moreover, AI-driven computational models can predict dopaminergic neuron loss, synaptic dysfunction, and disease spread more accurately than toxin-based PD models, which do not fully recapitulate the progressive nature of PD. Nonetheless, in vivo models are still required for the replication of complex interactions between the brain, immune system, and peripheral organs.

### Alzheimer’s disease

AD is the most common type of dementia, accounting for 60–80% of cases [138], and is clinically characterized by a progressive decline in memory and cognitive function, including visuospatial skills and executive functions [139]. The pathological hallmarks of AD include extracellular amyloid plaques formed by aggregation of Aβ oligomers into Aβ plaques and intracellular NFT caused by hyperphosphorylated tau, both of which gradually accumulate in the brain over several years. Aβ oligomers are considered the most neurotoxic species in AD since the levels correlate with the presence of synaptic loss and, in turn, cognitive symptoms, particularly during the earliest stages of the disease. In parallel, NFT accumulation is more strongly associated with neuronal and synaptic loss in moderate and advanced stages of the disease [140, 141].

Advanced age is an important risk factor in AD. The vast majority of cases initiate in individuals older than 65 years, known as late-onset AD. However, some cases present symptoms before the age of 65 years and are considered early-onset. Autosomal dominant mutations in presenilin 1, presenilin 2 (*PSEN1* and *PSEN2*), and the amyloid precursor protein (*APP*) genes account for about 10% of the familial AD cases (FAD) and typically is associated with early-onset. However, a few late-onset FAD cases have been reported [142–144]. The sporadic AD cases (SAD), with no known causative genetic mutations, account for 90–95% of all AD cases and are generally, although not exclusively, associated with late-onset AD [142, 143]. 

Although the genetic etiology and predispositions associated with AD greatly influence disease pathophysiology, the cause of the majority of FAD and SAD cases remains unexplained. Moreover, the critical impact of genetic and environmental risk factors of SAD, such as the apolipoprotein E4 (*APOE4*) allele, depression, diabetes mellitus, poor education, and loneliness, remain largely unknown [139]. Despite the overwhelming higher prevalence of SAD, 80% of AD preclinical research focuses on the use of transgenic mouse lines carrying mutations of FAD while only a few models use chemical induction, senescence, and cellular reprogramming from SAD patients [145]. 

### Animal models of AD

Various animal models of AD exploring specific features of the pathology have been reported over the years. Here, we provide a brief overview of the in vivo AD models that can be used to explore Aβ and tau co-pathologies as a step to inform directions to potential animal models of LBD (Table 3). A detailed review of animal models on AD pertaining species, AD pathogenesis, as well as features and limitations can be found in [146, 147].

**Table 3:** Animal models of Alzheimer’s disease

Model	FAD / SAD	Genetic mutation	Amyloid plaques	Neurofibrillary tangles	Synaptic impairment	Widespread neurodegeneration	Cognitive impairment	References
5xFAD transgenic mice	FAD	Swedish: APP^K670N/M671L^ Florida: APP^I716V^ London: APP^V717I^ PS1^M146L^ PS1^L286V^	High levels of intraneuronal Aβ42 beginning at ~ 2 months old Extracellular Aβ deposition begins around 2 months in the subiculum and cortex Plaques are found in the hippocampus and cortex at 6 months Older animals have plaques in the thalamus, brain stem, and olfactory bulb	No	Hippocampus: loss of synapses seen at 12 months	Cortical layer 5, subiculum, and the basal forebrain at 6 months Myelin abnormalities at 6 months of age. Parvalbumin-positive inhibitory interneurons were found in barrel fields of 12 months of age	Spatial memory impairment at 5 months in the Y-maze and 6 months in the water maze Conditioning fear tests impaired at 4 months Olfactory dysfunction at 6 months Motor impairments at 9 months Hearing impairments 14–16 months	[153] [259, 260]
3x Tg-AD transgenic mice	FAD	Swedish: APP^K670N/M671L^ PS1^M146L^ MAPT^P301L^	Intracellular Aβ is apparent at 4 months in the neocortex and by 6 months in the CA1 region of the hippocampus Extracellular Aβ deposits in layers 4 and 5 of the frontal cortex and hippocampus are apparent at 6 months	Observed at 18 months in the hippocampus	Occurs at 6 months in the hippocampus	Reduced neurons in the cortex at 11 months and in the CA1 region of the hippocampus in mice 12–15 month old mice	Impairments in retention in retrieval appear at 4 months 6.5 months mice display impairments in learning and memory in Barnes, Y- maze and fear conditioning	[161, 261, 262] [263]
APP NL-G-F transgenic mice	FAD	Swedish APP^K670N/M671L^ Iberian APP^I716F^ Arctic APP^E693G^	Plaques develop at 2 months with saturation by 7 months in homozygous mice AB deposition at 4 months in heterozygous mice	No	Yes at 3–4 months and severely impaired at 6–8 months in the CA1 region	No	Memory impairment by 6 months by the Y- maze	[159]
NFT transgenic mice	N/A	MAPT^P301L^ MAPT^P301S^	No	Accumulation in cell bodies at 3 months At 9 months the p-tau in the hippocampus resembles early-stage NFT of the human brain	No	Potentially	No	[160]
Chimeric mice models	FAD	Varies depending on the AD mouse model used	Aβ plaques have been observed in transplanted cells 4 months post-transplantation.	No	Reduction of dendritic staining around human neurons	Transplanted neurons undergo neurodegeneration (4 months post-transplantation), but the murine neurons do not.	Not reported	[163–165]
McGill-Thy1-APP rats	FAD	Swedish: APP^K670N/M671L^ Indiana: APP^V717F^	Extracellular plaques occur at 6 months By 16 months plaques are spread through the hippocampus and the cortex,	Yes	Reduction in synapse density seen at 20 months	Subiculum neuron loss at 22 months	Yes	[264]
Aged Chimpanzee	SAD	N/A	at ~ 35 years old plaques are found in cortical layers of the prefrontal cortex and medial temporal gyrus and in the CA1 and CA3 regions of hippocampus	at ~ 35 years old NFTs are observed in the CA1 of the hippocampus p-tau and Aβ co-occur in the hippocampus at ~ 35 years.	No	No	Chimpanzees begin to show cognitive decline around 30–45 years old	[265]
Aged Rhesus monkey	SAD	N/A	In the dorsolateral prefrontal cortex at 33–34 years old	Tau fibrils in the Entorhinal cortex in 24-26-year-old monkey Tau fibrils in pyramidal cells in 38-year-old monkeys p-tau in the dorsolateral prefrontal cortex at 31–34 years	May be induced by Aβ oligomers	In the entorhinal cortex layers at 33–36 years	26-year-old monkeys exhibit cognitive deficits	[169] [170, 171, 173]

Genetic and molecular mechanisms associated with AD have been modeled in *C. elegans* and *D. melanogaster*. Despite the lack of evolutionary complexity, *C. elegans* models maintain some synaptic transmission functions that can be interrogated in the context of Aβ overexpression, neurotransmitter signaling, and genetic risk factors such as expression of *APOE4* [148]. *D. melanogaster* models are also important because they allow for genetic manipulations that cannot be performed in mammals. For example, *D. melanogaster* γ-secretase-based models are useful to help elucidate the role and molecular mechanisms associated with mutations in the *presenilin* gene during development and degeneration [149]. Zebrafish present 84% homology to human dementia-related genes, including *APP*, *MAPT*, *PSEN1*, and *PSEN2.* [147] Additionally, zebrafish may exhibit AD-like cognitive and behavioral manifestations that can be further explored in drug screening to identify potential treatments for AD [150, 151]. 

Some of the most common mouse models of AD stemmed from the identification of specific mutations in the human *APP* gene, such as the Swedish (*APP*^K670N/M671L^) and Indiana (*APP*^V717F^) mutations, and in the *PSEN1* gene encoding presenilin 1, a component of γ-secretase, for example, the J20 mouse line (*APP*^K670N/M671L^ and *APP*^V717F^) and the APP/PS1 transgenic mice [146, 152]. Various APP/PS1 transgenic mouse models have been developed. Each model’s specific phenotype varies depending on the number and types of FAD mutations inserted and the promoters used. For example, while APP mutations may increase the accumulation of total Aβ or the aggregation-prone Aβ42, mutations in *PSEN1/2* alter the processing of APP without increasing accumulation [146]. The 5xFAD model expresses 3 mutations in the *APP* gene (*APP*^K670N/M671L^, *APP*^V717I^, and *APP*^I716V^) and 2 mutations in the *PSEN1* gene (*PS1*^M146L^ and *PS1*^L286V^) causing intracellular accumulation of Aβ as early as 6 weeks and plaque formation at 2 months [152, 153]. Typically, APP/PS1/2 mice models present significant Aβ aggregation with robust plaque formation, particularly in regions rich in plaques in AD brains such as the cortex and hippocampus. Alterations in the immune system, including astrocytosis and microgliosis, are moderately similar to those in AD [154–156]. Although a mild synaptic dysfunction potentially associated with subtle cognitive impairment in spatial tasks can be observed in some of these models, other AD pathological features are not recapitulated. Tau pathology, widespread neurodegeneration, and neurotransmitter abnormalities are absent. Moreover, the timing of cognitive impairment coincides with the early plaque formation in transgenic mice, instead of decades after plaque development in human AD [152, 157, 158]. The APP NL-G-F knock-in mice carry the Swedish (*APP*^K670N/M671L^), the Iberian (*APP*^I716F^), and the Arctic (*APP*^E693G^) mutations. The advantage of this transgenic mouse line over the previously discussed ones is that the APP NL-G-F knock-in mice overproduce Aβ42 with accompanying progressive Aβ pathology in an age-dependent manner without overexpressing APP. Thus, overproduction of other APP fragments is not present in APP NL-G-F mice. These animals also exhibit intense microgliosis and astrocytosis around Aβ deposits, with significant synaptic alterations that correlate with memory impairment at 6 months. The presence of the Arctic mutation accelerates the pathology relative to mice expressing only the Swedish and Iberian mutations (APP NL-F), leading to a more severe phenotype. NFTs and neurodegeneration are not detected in either mouse line [159]. 

Tau pathology is not observed in wild-type mice likely because the rodent tau has a different structure and sequence than the human tau (88% sequence homology) and may not be prone to aggregation [156]. Importantly, aggregation of human tau into NFT only occurs in mice lacking endogenous tau, showing that endogenous mouse tau inhibits the aggregation of human tau [160]. Robust NFT, neurodegeneration, atrophy, and motor deficits are typically achieved with transgenic overexpression of mutations on the *MAPT* gene (P301L, P301S) that cause frontotemporal lobar degeneration (FTLD) [115]. Despite intense tau pathology, these models do not represent the pathophysiology of AD given that these mutated forms of tau are not associated with AD and may interact differently with Aβ and change its toxicity. Moreover, the motor deficits observed in transgenic mice overexpressing mutated tau do not occur in AD [146, 152]. 

The 3xTg-AD model has become the most widely used AD model to study Aβ and tau co-pathology. It relies on the concurrent expression of mutated *APP*,* MAPT*, and *PSEN1* or *PSEN2*, for example, *APP*^K670N/M671L^, *PS1*^M146V^, and tau^P301L^ [161]. These transgenic mice initially develop intraneuronal Aβ accumulation followed by plaque formation in the cortex and hippocampus at 6 months. At this stage, minor neurodegeneration, synaptic impairment, and cognitive deficits can be observed. NFTs are formed at an older age (approximately 12 months) in the same brain regions presenting Aβ plaques [146, 162]. An important limitation of this model is the significant overexpression of mutated Aβ and tau which does not represent the majority of SAD cases.

Chimeric mouse models in which human iPSC-derived neuronal precursor cells and microglia have been exploited to better understand how human brain cells age and develop pathology in an in vivo system [163–165]. Espuny-Camacho and colleagues transplanted human neural precursor cells derived from pluripotent stem cells into a transgenic immunodeficient APP/PS1-21 mice model of AD [166] and observed that the xenografted human neurons respond to Aβ pathology differently than their murine counterparts. Particularly, Aβ plaques and Aβ-associated neuroinflammation were more pronounced around human transplanted cells and neurodegeneration was more abundant in human transplanted cells than in murine host cells [163]. Similarly, xenotransplantation of human stem cell-derived microglia in AD mouse models shows that human microglia respond differently than mouse microglia to Aβ pathology [164, 165]. A limitation of these models, however, remains the necessity to use immunocompromised mouse models, particularly in the study of immune responses to Aβ pathology.

There are only a few transgenic rat models of FAD. The McGill-R-Thy1-APP rat model, which carries the Swedish and Indiana mutations, closely recapitulates AD-like amyloid pathology and is the only model with extensive cognitive impairment characterization [146]. The TgF344 AD rat model is a double transgenic with the Swedish mutation and PS1ΔE9 shows strong accumulation of Aβ and NFT at 16 months despite the expression of only endogenous rat tau, not human tau [146, 162]. Although less popular than transgenic mice, the main advantages of transgenic rats involve better physiological and genetic similarities to humans. Additionally, larger bodies and brains make experimental approaches such as CSF collection, electrophysiology, and imaging easier, with a richer behavioral repertoire for more complex behavioral testing.

Non-human primates have long lifespans and can develop pathological and clinical manifestations highly similar to human AD, representing the most well-characterized SAD models [162]. Old age (~ 20 years) rhesus monkeys are the most common NHP SAD model because they present amyloid plaques in the cortex with an Aβ42/Aβ40 ratio similar to those observed in humans [146, 162]. Chimpanzees also accumulate Aβ in the brain, developing both amyloid plaques and congophilic amyloid angiopathy (CAA) in aged animals, but hardly develop tauopathy despite 100% sequence homology to human tau [167, 168]. Additionally, cognitive deficit resembles a mild cognitive impairment present in the early stages of human AD [146]. Accelerated pathology with widespread accumulation of Aβ, tau hyperphosphorylation, cholinergic dysfunction, synaptic loss, and glial activation was achieved with injection of Aβ oligomers in the lateral ventricles of middle-aged rhesus monkeys [169, 170]. Despite NHP models occasionally developing both Aβ and tau pathology, there are key differences to human AD that need to be considered. For example, rhesus monkeys tend to develop amyloid plaques in regional cortical areas (i.e., prefrontal lobe), whereas humans present plaques in the olfactory, frontal, parietal, and temporal cortices, hippocampus, and amygdala [169, 171, 172]. As in chimpanzees, NFTs rarely occur in rhesus monkeys [173]. 

### Cellular models of AD

Cellular models are an excellent resource for overcoming challenges inherent to animal modeling of AD, such as research focusing on FAD and confounding effects due to species differences. Primary cell cultures from rodents offer a good alternative for examining the pathological impact of Aβ and tau in cellular health and machinery but are restricted for not fully recapitulating the disease phenotype due to limited resemblance to age-related cellular immune dysfunctions [174, 175]. Moreover, post-mortem primary microglia isolated from AD patients rapidly lose disease-associated microglial phenotypes once removed from the brain microenvironment [176]. Cellular reprogramming of fibroblasts, blood cells, and urine-derived epithelial cells from FAD and SAD patient donors into iPSCs and then re-differentiation to neurons is advantageous because it allows for a detailed molecular examination of the disease pathophysiology and targeted therapeutic intervention [145, 146]. Notably, although iPSCs undergo extensive molecular changes during reprogramming and redifferentiation, it has been demonstrated that iPSC lines from AD donors continue to show increased ratio of Aβ42/Aβ40 and tau hyperphosphorylation in comparison to age-matched non-demented control iPSCs [177–180]. However, it must be emphasized that most of the iPSC AD lines have been generated from FAD donors which exhibit specific mutations culminating in specific phenotypes that might differ from SAD iPSC phenotypes [145, 178]. Moreover, genetic diversity can also affect experimental analysis as it may mask or exacerbate certain phenotypes.

Direct reprogramming of adult human fibroblasts from AD patients into neurons (iNs) poses an advantage to iPSC-derived neurons because it bypasses reprogramming the donor’s cells into the pluripotency stage. iPSC-associated rejuvenation erases age-associated or senescent phenotypes which are important risk factors in age-dependent diseases [129, 181]. In contrast, fibroblast-derived iNs maintain substantial signatures of human aging along with the pathological changes observed in neurons in AD brains [182–185]. Therefore, iNs from SAD patients represent a promising approach for studying age-related vulnerability and mechanisms relevant to AD that are not directly caused by genetic mutations or that have important genetic risk factors, such as *APOE4*.

The CRISPR/Cas9 system is an advantageous alternative to control genetic variances in a precise and reproducible manner by introducing or correcting specific mutations without altering the overall genetic background. For example, the introduction of AD-associated mutations in iPSCs from healthy donors or correction of mutations in iPSCs from SAD or FAD donors [186] offers a promising opportunity to minimally study the implications of single-point mutations [187]. 

A major limitation of iPSC and iN models is that the complexity of neuronal and glial interactions and the implications of such interactions to AD pathophysiology are not represented in a 2D cell culture. Microfluidic devices offer a promising solution to these challenges by maintaining the structural complexity of the central nervous system by allowing the integration of the BBB into neuronal and glial cells [188]. Three-dimensional cultures of multiple cell types also address this issue [145, 146, 188]. They can be made of a hydrogel or matrigel matrix that provides a flexible scaffold to sustain electrophysiological characteristics generated by the interactions between neurons and glia. They can also be made into different shapes to accommodate a wide range of applications. Studies have demonstrated that matrigel culture of differentiated human progenitor cells with FAD mutations presents aggregation and extracellular deposition of Aβ into plaques and tau hyperphosphorylation [189, 190]. 

Other 3D organoid models generated from human stem cells (human umbilical vein endothelial cells– HUVECs and human embryonic stem cells– hESCs) represent well-defined glial cells, astrocytes, and neurons that better mimic human cortical structure during development or during disease state. Recently, Chen and colleagues developed a SAD cortical organoid model from human iPSCs and exposed it to serum to mimic BBB breakage, a common AD feature, and observed increased Aβ-like pathology, hyperphosphorylated tau, synaptic loss, and an impaired neural network [191]. Additionally, Sun and colleagues successfully developed an iN-derived organoid model of non-genetic late-onset SAD patients using micro-RNAs (miR-9/9*+NEUROD2 + MYT1L) in a matrigel layer. The organoids were comprised of directly reprogrammed neurons and showed extracellular accumulation of Aβ, formation of seed-competent and insoluble tau, dystrophic neurites, and neurodegeneration (Table 4) [192]. Indeed, brain region-specific organoids, such as cortical organoids, midbrain organoids, and hippocampal organoids, hold the promise to open a vast horizon of new research possibilities given the intricate 3D organization of cellular interactions combined with the extracellular deposition of pathological proteins. Therefore, organoid cultures enhance our capability to establish patient-specific models based on genetic elements and the potential for a targeted therapeutic approach [193]. 

**Table 4:** Cellular models of Alzheimer’s disease

Cell type	FAD/SAD	Amyloid aggregates (Aβ plaques)	Hyperphosphorylated tau (NFT)	Synaptic dysfunction	Cell death	References
iPSC (patient-derived induced pluripotent stem cells)	FAD or SAD	Yes, Aβ42 and APP levels increase 42 days past differentiation in FAD and SAD cell lines.	Yes, elevated phosphorylation observed at 42 days post differential date. Peak at 52 to 70 days after differentiation.	Potentially	No	[194] [195]
Brain organoids from patient-derived iPSCs	FAD or SAD	Yes, 12 days after serum treatment in SAD models. Yes, it is present in FAD genetic mutation organoids.	Yes, 12 days after serum treatment SAD models. Yes, it is present in the FAD genetic mutation organoids.	In *APOE* and *APP/PSEN1* organoids there is a decrease in synaptic integrity. Yes, in serum-treated SAD models.	*APOE4* increases neurodegeneration in iPSC-derived organoids, especially in deeper layers.	[191] [193]
Patient-derived iNs (direct reprogrammed induced neurons)	FAD or SAD	Yes, Aβ42 levels increase in FAD INs. *APOE4* genotype treated with APP.	FAD fibroblasts do not show elevated levels of tau. *APOE4* genotype treated with APP.	Potentially	No	[181, 182, 184, 185, 196]
Brain organoids from patient-derived iNs	FAD or SAD	Yes, in FAD models with *APP* and *PSEN1* mutations. SAD models show increased Aβ deposition.	Yes, in FAD models there is an increase in phosphorylated tau and spherical beads in neurites. SAD models show increase of phosphorylated tau.	SAD models show impairment in synaptic formation.	FAD models have increased cell death compared to healthy controls. SAD models show neuronal loss and neurite deposition in cortical neurons.	[192]

### In Silico models of AD

In silico models for Alzheimer’s disease leverage computer simulations to replicate aspects of the disease, simplifying complex biological systems into manageable models with a minimal number of parameters. These models often draw from structural insights provided by in vitro experiments, resulting in a close interplay between the two methodologies.

The process of protein oligomerization to form fibrils and plaques can be modeled in silico by determining the structures and interaction forces that govern each step [197]. This is essential for screening and identification of binding sites and specific ligands capable of inhibiting the fibrillization process [198]. The combination of cryo-electron microscopy, solid-state nuclear magnetic resonance, computational 3D mapping, and atomic modeling allowed the reconstruction of the structure of recombinant Aβ42 fibrils generated from *E. coli* [199]. Interestingly, the structural analysis of Aβ fibrils isolated from meningeal tissues of AD patients showed discrepancies from the synthetic fibrils and variations correlated to clinical AD phenotype, indicating the possibility of different Aβ strains [200]. 

Computer simulations are also used to screen for aggregation inhibitors in a resource and timesaving manner [201, 202]. Nie and colleagues used MDS to demonstrate the molecular recognition mechanisms of Aβ40 monomers and gallic acid, a natural polyphenol inhibitor of Aβ fibrillization, which helps elucidate the anti-amyloidogenic effect of polyphenols [203]. 

AI-driven modeling methods are promising tools to integrate neuroimaging, genomics, and clinical data to predict AD progression, optimize diagnostic accuracy, and tailor personalized therapies [204, 205]. AI can enhance the analysis and interpretation of human-relevant models, such as patient-derived organoids, which more accurately reflect human AD pathology than transgenic animal models. Additionally, AI simulations can be used to predict the specific contributions of genetic mutations (e.g., *APP*, *PSEN1*, *APOE4*) to Aβ and tau pathology, potentially reducing the need for transgenic mouse models. These strategies will improve our understanding of network and molecular changes associated with neurodegeneration in AD.​.

### Lewy body dementia

LBD is the third most common dementia after AD and vascular dementia. Like most neurodegenerative diseases, age is the strongest driver of developing LBD with increased risk over 60 years old [206]. LBD is characterized pathologically by the widespread occurrence of LP. While brainstem LP and dopaminergic neuron loss in the SNc are hallmarks of PD, LBD cases exhibit a more widespread distribution of pathology and degeneration, with the limbic system and neocortex being affected in addition to the brainstem. Additionally, LBD cases often exhibit concomitant AD-related pathologies including Aβ plaques and, to a lesser extent, NFTs [207]. The progression of AD-related pathologies follows a subcortical to neocortical route, beginning in the entorhinal and hippocampal regions and fanning outwards as the disease progresses. In contrast to AD, however, hippocampal atrophy is far less pronounced in LBD [208]. PD pathology progresses in a caudal-rostral manner, thought to be a result of the spread and seeding of pathological αsyn through interconnected neuronal circuits [209–211]. 

Attributed to the distribution and confluence of pathologies, LBD patients may exhibit AD-like dementia symptoms and classical parkinsonism, as well as certain distinguishing cognitive impairments including visual hallucinations, cognitive fluctuations, and neuroleptic sensitivity [206]. While arbitrary, the one-year rule for the manifestation of Parkinsonism vs. cognitive symptoms helps clinicians stratify PDD and DLB patients and contextualize disease progression. At late stages of the disease, however, the clinicopathological features of PDD and DLB often look indistinguishable. The distinction between PDD and DLB also holds relevance when discussing the translational relevance of LBD models. Some models may be more in line with PD pathology and symptomatology while others may primarily represent AD characteristics.

In addition to overlapping clinicopathological features, LBD also shares many genetic risk loci and variants with AD and PD [207, 212]. For example, *APOE4* and *GBA* are the strongest risk factor genes for LBD as well as AD and PD, respectively. Other LBD risk factors include *APP*, *SNCA*, *PARK2*, *MAPT*, and many others with disease overlap. These features hint that LBD may represent an important disease state that bridges AD and PD through overlapping mechanisms involving Aβ, αsyn, and tau proteinopathies. Given the diversity and heterogeneity of genes underlying LBD etiology, most models rely on AD and PD familial mutations along with frontotemporal dementia mutations in *MAPT*, to drive amyloid, αsyn, and tau pathologies.

### Animal models of LBD

Many of the PD models described in this review also model key aspects of “pure” synucleinopathy LBD. However, since most LBD cases involve a confluence of αsyn, Aβ, and tau pathologies, the focus of this section will be on reported mixed-pathology animal models of LBD. To date, no mixed pathology model has been developed in non-mammalian species, such as *C. elegans* and *D. melanogaster*. These models, although evolutionary simple, are useful tools to enhance our understanding of the basic mechanisms underlying causal genes of AD and PD, protein-protein interactions between αsyn, Aβ, and tau, and as a pharmacological screening approach.

The development of mixed models can be put into three general categories: transgenic crosses, transgenics plus PFF inoculations, and transgenics plus viral AAV-mediated transgene expression. Within each category, there have been several different mouse lines, PFF species, and AAVs leveraged or developed, each with their own strengths and caveats– some supporting the same conclusions, others providing divergent results (Table 5). Assessing how they match up to the human condition is necessary for attributing their value to the field.

**Table 5:** Animal models of Lewy body dementia

Model	Amyloid pathology	Tau pathology	α-synuclein pathology	Synaptic deficit	Dopaminergic deficit	Widespread neurodegeneration	Cognitive impairment	Motor impairment	Potential Synergism	References
hAPP/hSYN (hSYN line D x hAPP line J9) mice	Aβ deposition unchanged by synuclein	Not reported	Increased and more fibrillar (15%) αsyn accumulation over time	Loss of synaptophysin terminals (4–20 months)	Not reported	Cholinergic neurodegeneration (4–20 months)	Memory deficits driven by hAPP (6 months)	Motor deficits accelerated by hAPP (6 months)	Yes, Aβ enhances αsyn aggregation, neurodegeneration, and motor deficits	[213]
DLB-AD (3xTg-AD x M83-h A53T syn) mice	Increased Aβ plaques (6–12 months)	Increased tau tangles (6–12 months)	Increased αsyn inclusions (6–12 months)	Significant synaptic loss (6–12 months)	Not reported	No difference in 3xTg vs. DLB-AD	Accelerated cognitive decline (6–12 months)	Not detected	Yes, synergistic interactions exacerbated all 3 pathologies	[3]
APP/PS1 x [A30P] aSYN mice + αsyn PFF or ampLB	Reduced Aβ plaques 4–16 weeks	Not reported	Not detected	Not reported	Not reported	Not reported	Preserved cognitive function (up to 12 months)	Not reported	No, αsyn inhibited Aβ aggregation	[219]
APP J20 x αsyn Tgl2.2 (APP/αsyn); APP/αsyn-KO mice	Reduced Aβ plaques with αsyn overproduction, increased Aβ with αsyn knockout (up to 12 months)	Increased MC1 & CP13 immunoreactivity in APP/αSyn mice (6 months)	Increased in αsyn oligomers in APP/αsyn mice (6 months)	Synaptic markers decreased in APP/αsyn mice (6 months)	TH + neuron loss in SNpc and VTA, 4.5 months 6mpi	Not reported	Worsened memory with αsyn overproduction; improved with knockout (6 months)	Not reported	No, bidirectional effects of αsyn and Aβ observed	[220]
5xFAD mice + αsyn mPFF APP-KI mice + αsyn mPFFs	Increases in 1.5 months injected mice at 6mpi, increases in 4.5 months injected at 3 and 6mpi	neuritic at 3 mpi neuronal and neuritic at 6 mpi	Increase in brain-wide αsyn pathology from 3–6 mpi	dystrophic neurites	Not detected	NeuN + neuron loss in the hippocampus	Y maze by 3mpi	Motor deficits in rotarod 3 mpi	Yes, Aβ promotes seeding of αsyn and tau	[217]
APP/PS1 L85 x h-αsyn M20 (M20/L85) mice + αsyn PFF	PFFs increase Aβ in APP mice but not αsyn/APP mice	Not reported	Aβ exacerbates αsyn pathology (2–4 months post-injection)	Not detected	Not reported	Not reported	Not reported	Not reported	Yes, Aβ exacerbates αsyn pathology in APP/αsyn mice, and αsyn PFFs exacerbate Aβ in APP mice	[218]
hThy1-αsyn “Line 61” mice + AAV-tau; hTau mice + AAV-αsyn; APP/PS1 mice + AAV-αsyn	APP/PS1 pathology unaffected by AAV-αsyn production at 3 months post-injection (6 months)	hTau pathology mild/ unaffected by AAV-αsyn at 6 mpi (9 months)	Thy1-αsyn pathology mild and unaffected by AAV-tau expression 6 mpi (9 months)	Not reported	Not reported	Not reported	Motor and cognitive impairments in base animal models not affected by additional transgene delivery via AAV	Not reported	No, additional transgene delivery via AAV did not exacerbate any existing pathologies or behaviors	[221]

The seminal example of a mixed-pathology amyloid and αsyn model is the hAPP/hSYN mouse [213]. It is a double transgenic cross of the previously characterized mutant hAPP J9 line [214] and wild-type hSYN D line [213, 215]. The phenotypes of hSYN-only mice are relatively mild, presenting moderate and non-fibrillar αsyn inclusion formation and deficits in tyrosine hydroxylase and motor balance at 12 months old. The addition of hAPP in hAPP/hSYN mice bumps the motor phenotype up to 6 months causing a stronger age-dependent accumulation of αsyn inclusions with more fibrillar species detected. Additionally, at 20 months old, more substantial cholinergic neuron and synapse loss is observed in hAPP/hSYN mice than in hAPP-only mice [213, 215]. Interestingly, while hAPP was found to increase hSYN-related pathology, hSYN expression did not alter amyloid plaque pathology or associated neuritic dystrophy. Overall, the data suggest that hAPP drives the synergistic interactions between αsyn and APP in this model. The purported directionality of the relationship could be attributed to the fact that hAPP was overexpressed, while hSYN was not, perhaps causing APP’s products to dominate the phenotypes measured. Indeed, a transgenic αsyn/amyloid/tau pathology mouse line (DLB-AD), established by crossing the 3xTg-AD [161] amyloid/tau line with the M83-h [216] mutant A53T αsyn line, demonstrated that αsyn pathology is capable of promoting Aβ and tau accumulation [3]. Relative to their 3xTg and M83 counterparts, DLB-AD mice display significantly higher phosphorylated αsyn levels at 6 and 12 months, increased Aβ and phosphorylated tau levels by 12 months, and accelerated cognitive decline. Notably, motor function in the rotarod test and inflammatory glial responses between DLB-AD and 3xTG mice remain unchanged at any age [161]. While the evidence from the DLB-AD and hAPP/hSYN transgenic mice supports a synergistic relationship amongst LBD-related pathologies, the directionality of such pathologies is likely model-dependent.

In support of synergistic interactions, Bassil and colleagues demonstrated that the unilateral hippocampal injection of mouse αsyn PFF (mPFF) into the aggressive 5xFAD amyloid model leads to more severe and widespread αsyn pathology, hyperphosphorylated tau detection, hippocampal and midbrain neuronal loss, and cognitive and motor deficits [217]. While an AD overexpression transgenic model combined with a PFF model is poised to implicate the transgene as the main driver of observed synergism, the authors showed that mPFF increased Aβ plaque area in the hippocampus. Similarly, human αsyn (M20) and APP/PS1 (L85) double transgenic mice showed that the presence of Aβ plaques exacerbated αsyn aggregates and neuroinflammation triggered by human αsyn PFF injection in the hippocampus. Surprisingly, PFF injection into L85 mice increased Aβ deposition without eliciting the same effect in the L85/M20 mice [218]. This demonstrates that directionality is driven by singular factors in different models.

Conversely, other studies suggest that amyloid/αsyn interactions may have a protective effect on Aβ pathology. Bachhuber and colleagues demonstrated that a variety of αsyn-containing homogenates or PFFs prevented Aβ deposition in APP/PS1 mice between 6 and 16 weeks old [219]. Similarly, a reduction in amyloid plaque load was observed in 4-month-old APP/PS1 x [A30P]αSYN dTg mice compared to their APP/PS1 littermates. It is possible there is an initial protective effect of αsyn on Aβ given the relatively young age of the mice. As mice age, such protective effect may be lost due to higher pathology burden. Another model supporting a protective role of αsyn on amyloid burden was reported by Khan and colleagues using a novel bigenic APP/αsyn (APP J20/Tgl2.2) mouse at 6 months old [220]. Interestingly, despite the reduction in amyloid burden, these mice presented more cognitive deficits than their singly transgenic counterparts. However, αsyn ablation in APP/αsyn-KO mice caused increased amyloid burden and rescued APP-driven cognitive deficits. Recent efforts by Lim and colleagues, aimed to elucidate mixed-pathology interactions by using a transgenic plus viral-mediated gene delivery approach, whereby they injected adult Line 61 (hThy1-αsyn) mice with AAV-tau, hTau mice with AAV-αsyn, and APP/PS1 mice with AAV-αsyn. Despite achieving brain-wide wild-type human tau and αsyn production via AAVs, the resulting pathologies were low and did not affect the behavioral outcomes nor interacted with the genotype-driven pathologies [221]. 

### Cellular models of LBD

Cellular models of LBD primarily serve to elucidate and validate mechanisms of pathogenesis by replicating key disease features, such as the interactions between αsyn, Aβ, and tau. These models help characterize pathological mechanisms and establish relationships between genetic mutations, protein aggregation, and cellular dysfunction, providing a controlled environment to confirm hypotheses about disease progression. Cellular models of LBD may include LBD patient-derived systems, such as dopaminergic, cholinergic, and pyramidal neuron monocultures, mixed cell type co-cultures, and 3D organoid structures. Alternatively, using CRISPR/Cas9 gene editing strategies described previously, LBD-associated risk variants (e.g. A152T-MAPT) or causal mutations (e.g. E46k-*SNCA*; *SNCA* triplication) can be introduced into control iPSC lines to promote consistency and establish causality. Alternatively, immortalized neuron-like cell lines and primary neuron or neuron-glia co-cultures from mixed-pathology transgenic rodents can be used. α-Synuclein and tau PFFs are frequently used in cellular models to seed robust intraneuronal pathologies that mirror in vivo findings while amyloid pathology requires recombinant Aβ fibrils or oligomers treatment. Viral-mediated overexpression of transgenes is another commonly used method in cellular models given its flexibility, modularity, and robustness.

Concrete examples of in vitro research of LBD pathophysiological mechanisms are still scarce. The first attempt to represent LBD in a cellular model was made by Masliah and colleagues in 2001. They showed that treatment of GT1-7 hypothalamic neuronal cells with synthetic Aβ42 promoted intracellular accumulation of αsyn inclusions and suggested that the effect is likely downstream of Aβ peptide intracellular uptake, which could be distinct from effects of extracellular plaques in vivo [213]. Several years later, in 2015, Bachhuber et al. used primary neurons from APP/PS1 x [A30P] αSYN transgenic mice to demonstrate that αsyn overproduction does not affect extracellular Aβ release. This is possibly due to the inhibition of amyloid plaque formation through an extracellular interaction between the proteins or a neuron-extrinsic effect [219]. Recently, Jin and colleagues described a novel patient-derived *SNCA*-triplication cortical organoid model of LBD that exhibits aggregated αsyn, mitochondrial dysfunction, and metabolic and synaptic pathway dysregulation [222]. Importantly, Aβ42 and tau levels were unchanged in this model indicating the highly elevated αsyn production does not promote co-pathologies in the timeframe studied. Building from approaches discussed for PD and AD models, this LBD organoid model could be combined with viral or PFF-mediated tau or amyloid pathology to create a platform targeting co-pathologies interactions.

### In Silico models of LBD

LBD in silico modeling is still in its early stages, but it holds promise for advancing our understanding of the disease and developing effective therapies. Considering the current progress of in silico PD and AD modeling platforms, we anticipate that MDS studies exploring the behavior of αsyn, Aβ, and tau at the molecular level will provide insights into the aggregation process, protein-protein interaction, and interactions with cellular components. Network-based models representing neural networks and their connectivity will help elucidate how αsyn aggregates spread across different brain regions with and without Aβ and tau co-pathologies. Large-scale proteomic data integration will allow the identification of LBD biomarkers to facilitate personalized medicine strategies by highlighting individual differences in disease manifestation and progression. Additionally, QSP frameworks (pharmacokinetics and pharmacodynamic models) simulating how drugs interact with biological systems in the context of LBD will aid in dosing regimens optimization and therapeutic outcomes prediction. Ongoing research and technological advancements are expected to enhance the sophistication and applicability of these models in the near future. For this, collaborative efforts to share clinical and experimental data are essential to overcome the scarcity of comprehensive datasets specific to LBD. AI can accelerate the modeling of LBD by simulating αsyn, Aβ, and tau pathologies based on patient-derived data or cell-based models. Its ability to integrate complex datasets allows for more precise, scalable, and personalized approaches to understanding and treating LBDs. Lastly, ensuring that computational models accurately reflect biological reality will require rigorous validation against experimental and clinical findings. This step is crucial for the models to be reliable tools in research and therapeutic development.

### Current challenges and future directions for LBD models

LBD modeling presents unique challenges due to the complex and multifaceted nature of the disease. Capturing the heterogeneity of LBD pathology in vivo and in vitro remains a challenge. Although useful tools for specific molecular, cellular, or behavioral questions, in vitro and in vivo LBD models do not fully recapitulate αsyn pathology, the aggregation process, biochemical features, propagation mechanisms across brain regions, and behavioral dysfunctions. The use of cell lines overproducing αsyn or PFF-treated may not accurately reflect physiological processes. Additionally, simplified in vitro systems do not capture the full cellular diversity or the interactions between neurons and glial cells, such as astrocytes and microglia, which play a key role in LBD pathology. Similarly, differences in immune responses, neuronal networks, and αsyn biology between animal models and humans limit translational potential.

Efforts to improve models of LBD should aim to create systems that more accurately replicate human disease, paving the way for effective diagnostics and therapies. Brain organoids and patient-derived 3D cultures from either iPSCs or direct reprogramming can better mimic the cellular diversity and architecture of the human brain [223]. These models allow for studying neuron-glia interactions and disease progression in a more physiologically relevant context. Microfluidic devices can simulate the connectivity between different brain regions, enabling studies on the spread of protein aggregates [224]. Additionally, high-throughput screening platforms can accelerate the discovery of therapeutic compounds targeting mixed pathology. However, important limitations to organoid models can compromise their ability to model late-stage disease processes. Lack of vascularization, which restricts nutrient and oxygen diffusion, can lead to hypoxia and necrotic cores in larger structures. For this reason, smaller organoid systems may be preferred at the expense of low cellular diversity, loss of long-range connectivity, and electrophysiological properties that do not fully recapitulate disease states [225, 226]. 

**Fig. 1:**
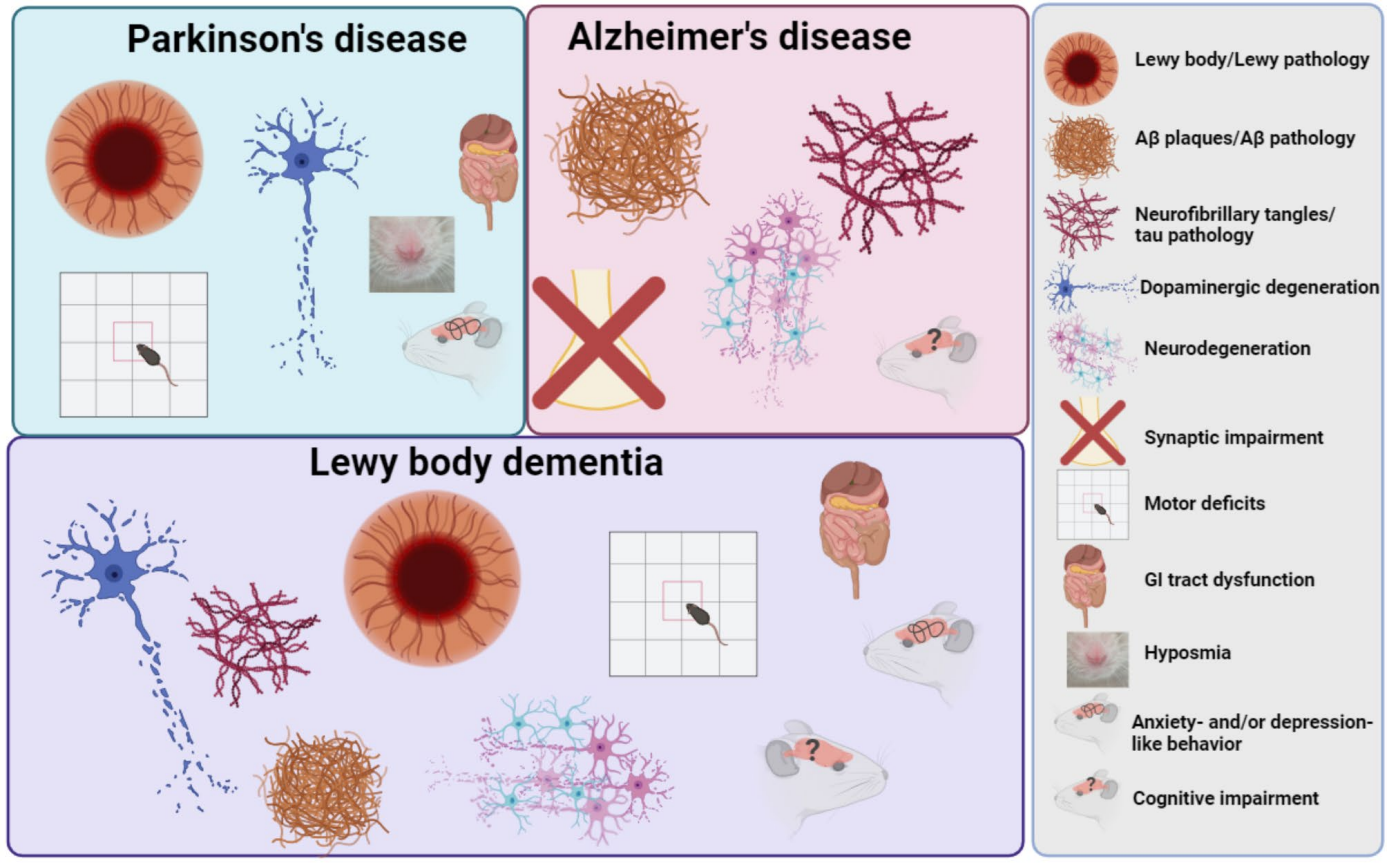
Pathological characteristics in PD, AD, and LBD models. Summary of key pathological and symptomatic features expressed in animal models of PD, AD, and LBD to ideally recapitulate human conditions with high construct, face, and predictive validities

The main focus of LBD in vivo modeling is to create humanized animal models that integrate genetic, environmental, and aging factors to better mimic the multifactorial nature of LBD (Fig. 1). Improved methods and technology to generate animal lineages that express human-specific PD and AD risk genes combined with the development of sensitive tools to measure cognitive and motor impairments will enhance their translational value.

Modeling LBD in NHPs holds significant value due to their close genetic, anatomical, and functional similarities to humans [227]. NHPs develop motor deficits (e.g., bradykinesia, rigidity, and tremors) that closely mirror the Parkinsonian features also seen in PDD and DLB. These deficits are often challenging to replicate in rodent models. Similarly, NHPs exhibit complex behaviors and cognitive processes, such as working memory, executive function, and visuospatial abilities, that can be assessed using well-established neuropsychological tests [228]. Additionally, NHPs provide opportunities to identify and validate biomarkers, such as neuroimaging changes (PET/MRI) and CSF or blood-based αsyn levels, which are critical for monitoring disease progression and evaluating therapeutic responses [229]. 

While NHP models provide unparalleled translational value, there are notable challenges regarding the expensive costs required for specialized facilities and care of NHPs [230]. As discussed in AD NHP models, LBD is a chronic, progressive disease, and modeling it in NHPs requires long experimental timelines. Finally, ethical considerations regarding the use of NHPs in research necessitate stringent regulations and justification for their use, which represents a barrier to LBD NHP modeling for many institutions [230]. 

### Concluding remarks

The choice of an optimal model system depends on a balance between the main scientific question and the strengths and limitations of the particular model. The experimental design must be carefully developed to maximize the strengths and minimize the limitations of the model so that its translational validity is properly addressed. Traditional PD and AD cellular models based on immortalized cells and primary neurons as well as animal models based on neurotoxin-induced lesion and viral-mediated transgene overexpression are well characterized, widely accepted, and provide relatively straightforward methods to study disease mechanisms and potential therapeutics. Overall, they hold good predictive validity and somewhat acceptable face validity. However, they lack etiological and construct validities, which encourages a focus on the development of translational models that more closely mimic the pathogenesis and pathological mechanisms of the disease. Therefore, PD and AD patient-derived iPSC cultures in a 2D or a 3D system, as in organotypic cultures, are highly relevant at the current stage of neurodegeneration research.

In LBD research, the pathogenesis and pathophysiological mechanisms of the human clinical condition are still obscure. For this reason, cellular and animal models are essential tools to validate each other’s findings and to provide opportunities for LBD research advancement. The conclusions from the animal and cellular models exploring the dynamics of the mixed protein pathologies illustrate the complex relationships between Aβ, tau, and αsyn in LBD. While most models support the idea that these proteins interact to exacerbate neurodegeneration, there are notable exceptions where αsyn appears to have a protective role. As research continues, it is critical to refine these models, exploring the precise mechanisms underlying these interactions, their relevance to human disease, advance NHP LBD models, and develop multi-targeted therapies for the multiple proteinopathies existent in LBD.

Progress in LBD research models will be achieved with continued multidisciplinary collaboration across bioinformatics, neuroimaging, molecular biology, and systems neuroscience. Incorporation of in silico models to simulate disease dynamics and optimize experimental design is critical for in vitro and in vivo studies so that the combination of organoid models with animal studies can provide complementary insights, bridging the gap between mechanistic research and clinical applications.

## Data Availability

Data sharing is not applicable to this article as no datasets were generated or analyzed during the current study.

## Abbreviations

6-OHDA: 6-hydroxydopamine
AAV: Adeno-associated virus
AD: Alzheimer’s disease
AI: Artificial intelligence
ampLB: Alpha-synuclein aggregates amplified from patient-derived Lewy bodies
Aβ: Amyloid beta
BBB: Blood-brain-barrier
CAA: Congophilic amyloid angiopathy
CSF: Cerebrospinal fluid
DA: Dopamine
DLB: Dementia with Lewy body
FAD: Familial Alzheimer’s disease
FTLD: Frontotemporal lobar degeneration
GWAS: Genome-wide association studies
iDAN: Induced dopaminergic neuron
iN: Induced neuron
iPSC: Induced pluripotent stem cell
LB: Lewy body
LBD: Frontotemporal lobar degeneration
FTLD: Lewy body dementia
LN: Lewy neurite
LP: Lewy pathology
LUHMES: Lund human mesencephalic cells
MDS: Molecular dynamics simulations
MFB: Medial forebrain bundle
Mpi: Months post-injection
MPTP: 1-methyl-4-phenylpyridinium
NFT: Neurofibrillary tangles
NHP: Non-human primate
PD: Parkinson’s disease
PDD: Parkinson’s disease dementia
PFF: Pre-formed fibrils
QSP: Quantitative systems pharmacology
ROS: Reactive oxygen species
SAD: Sporadic Alzheimer’s disease
SNc: Substantia nigra pars compacta
UPRS: Unified Parkinson’s Disease Rating Scale
αsyn: Alpha-synuclein
